# Supplementary material for: Selenocysteine Machinery Primarily Supports TXNRD1 and GPX4 Functions and Together They Are Functionally Linked with SCD and PRDX6
Source: Biomolecules. 2022 Jul 28;12(8):1049. doi: 10.3390/biom12081049 (PMC9405853; doi:10.3390/biom12081049)
Supplement: Supplementary file 1 [file biomolecules-12-01049-s001.zip › biomolecules-1799838-supplementary.pdf]

Supplementary figures

Selenocysteine machinery primarily supports TXNRD1 and GPX4 functions and together they are functionally linked with SCD and PRDX6

Didac Santesmasses[1] and Vadim N. Gladyshev[1,*]

[1] Division of Genetics, Department of Medicine, Brigham and Women's Hospital, Harvard Medical School, Boston, MA
* Correspondence: vgladyshev@rics.bwh.harvard.edu

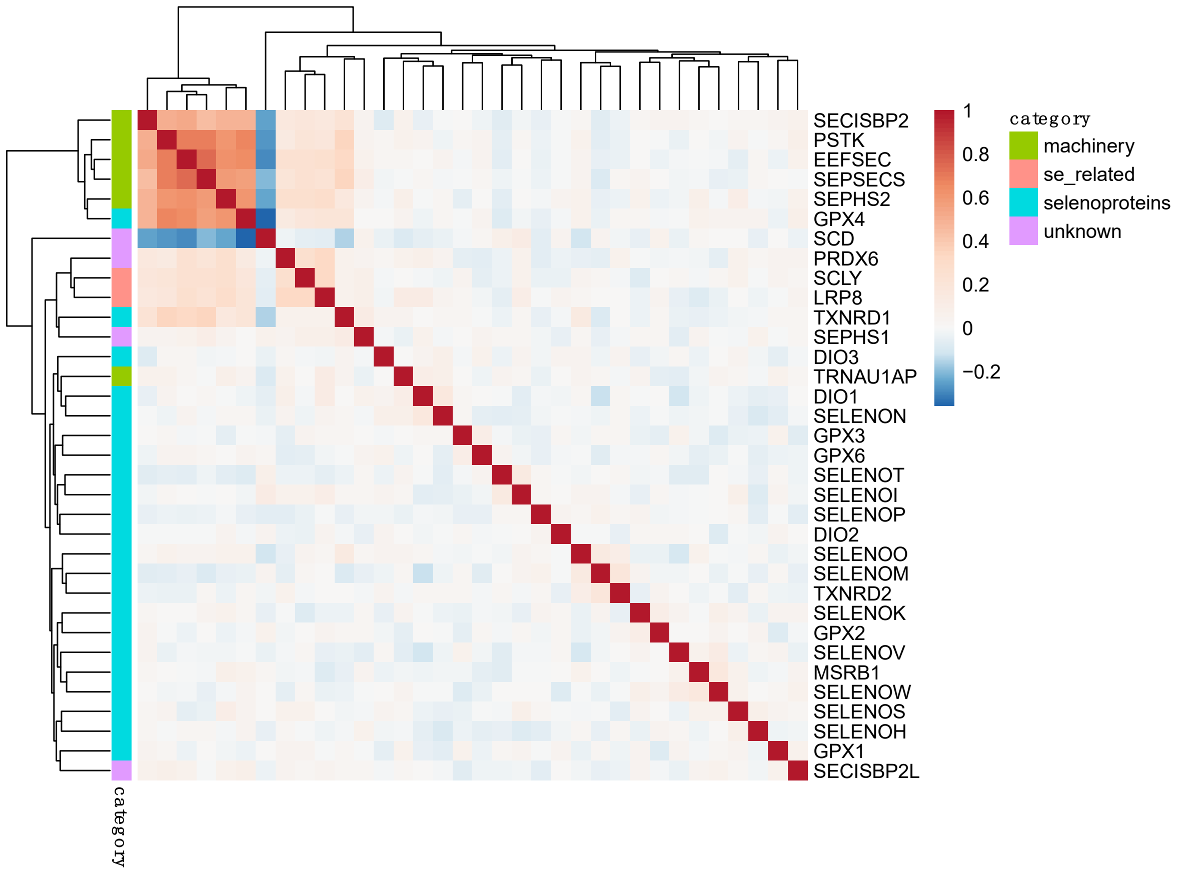

Figure S1. Correlation heatmap of cell viability scores between selenoproteins and proteins in the Sec cluster. Values correspond to Pearson's correlation across 941 cancer cell lines from the Cancer Dependency Map.

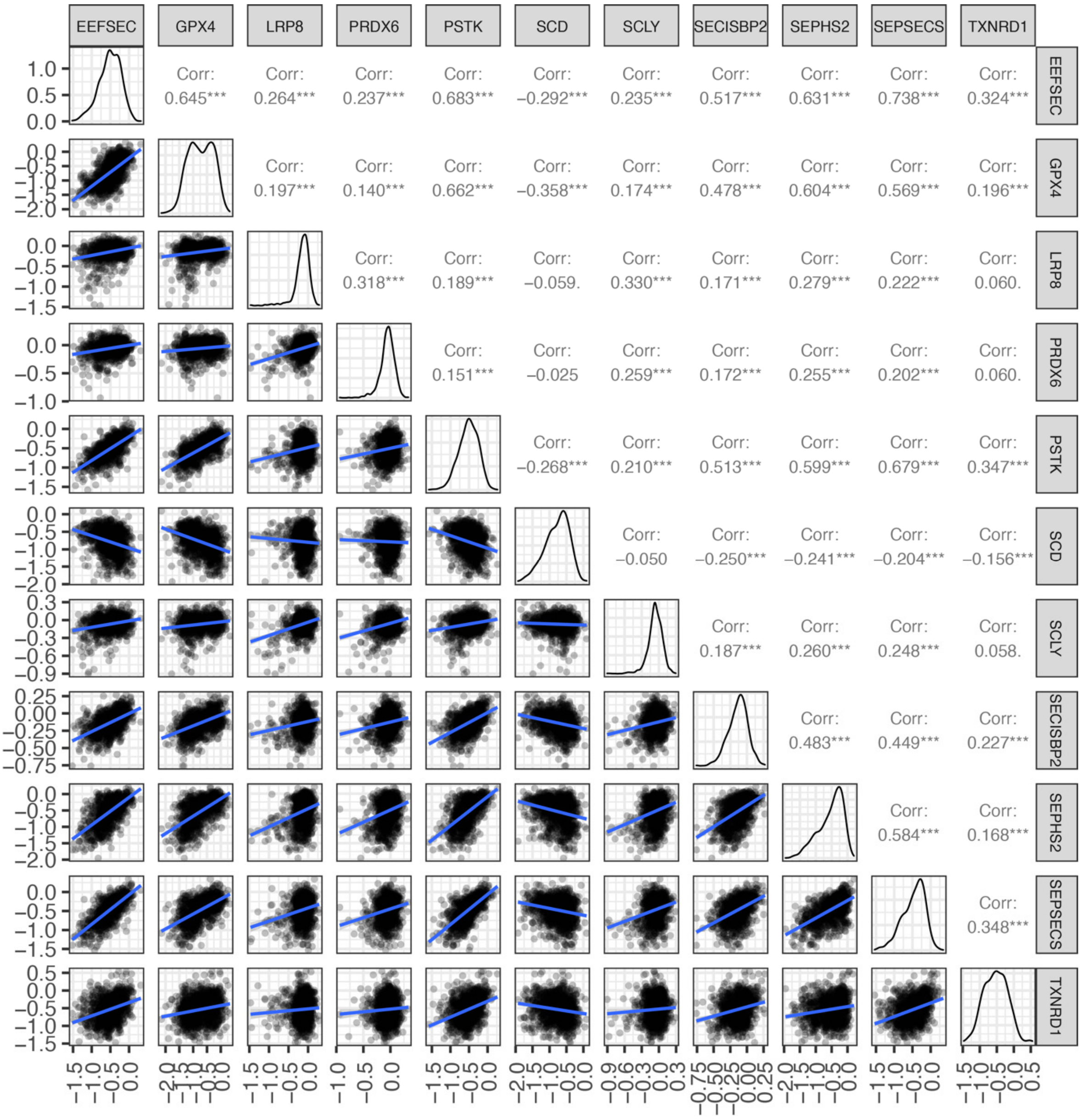

Figure S2. Pairwise correlation of essentiality scores across 941 cancer cell lines for genes in the Sec cluster. Bottom left corner shows the scatter plot and fitted linear model between the corresponding pair of genes, and the top right corner shows Pearson's correlation coefficient and statistical significance level.

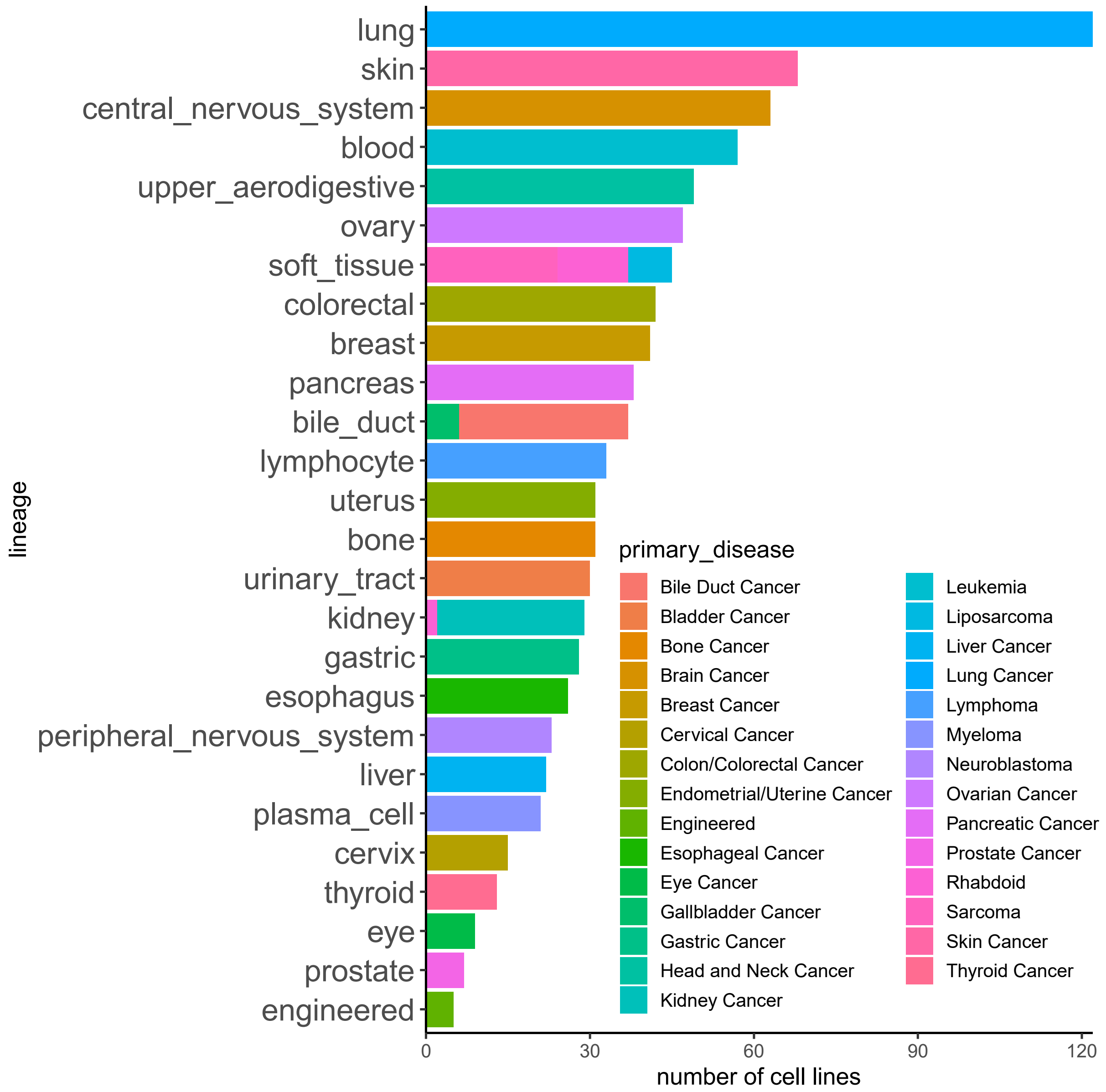

Figure S3. Number of cell lines per lineage (primary tissue), colored by primary disease, in the Cancer Dependency Dataset.
